# Challenges Experienced by Health Care Workers During Service Delivery in the Geographically Challenging Terrains of North-East India: Study Involving a Thematic Analysis

**DOI:** 10.2196/57384

**Published:** 2024-09-10

**Authors:** Sumit Aggarwal, Simmy Simmy, Nupur Mahajan, Kuldeep Nigam

**Affiliations:** 1 Indian Council of Medical Research New Delhi India

**Keywords:** challenges, thematic analysis, infrastructure, communication, supply distribution, resilience, adaptability, vaccination awareness, innovative solutions

## Abstract

**Background:**

The public health landscape in North-East India is marked by the foundational principle of equitable health care provision, a critical endeavor considering the region’s intricate geography and proximity to international borders. Health care workers grapple with challenges, such as treacherous routes, limited infrastructure, and diverse cultural nuances, when delivering essential medical services. Despite improvements since the National Rural Health Mission in 2005, challenges persist, prompting a study to identify health care workers’ challenges and alternative strategies in Manipur and Nagaland.

**Objective:**

This study aims to document the challenges experienced by health care workers during service delivery in the geographically challenging terrains of North-East India.

**Methods:**

This study is part of the i-DRONE (Indian Council of Medical Research’s Drone Response and Outreach for North East) project, which aims to assess the feasibility of drone-mediated vaccine and medical delivery. This study addresses the secondary objective of the i-DRONE project. In-depth interviews of 29 health care workers were conducted using semistructured questionnaires in 5 districts (Mokokchung and Tuensang in Nagaland, and Imphal West, Bishnupur, and Churachandpur in Manipur). Nineteen health facilities, including primary health care centers, community health centers, and district hospitals, were selected. The study considered all levels of health care professionals who were in active employment for the past 6 months without a significant vacation and those who were engaged in ground-level implementation, policy, and maintenance activities. Data were recorded, transcribed, and translated, and subsequently, codes, themes, and subthemes were developed using NVivo 14 (QSR International) for thematic analysis.

**Results:**

Five themes were generated from the data: (1) general challenges (challenges due to being an international borderline district, human resource constraints, logistical challenges for medical supply, infrastructural issues, and transportation challenges); (2) challenges during the COVID-19 pandemic (increased workload, lack of diagnostic centers, mental health challenges and family issues, routine health care facilities affected, stigma and fear of infection, and vaccine hesitancy and misinformation); (3) perception and awareness regarding COVID-19 vaccination; (4) alternative actions or strategies adopted by health care workers to address the challenges; and (5) suggestions provided by health care workers. Health care workers demonstrated adaptability by overcoming these challenges and provided suggestions for addressing these challenges in the future.

**Conclusions:**

Health care workers in Manipur and Nagaland have shown remarkable resilience in the face of numerous challenges exacerbated by the pandemic. Despite infrastructural limitations, communication barriers, and inadequate medical supply distribution in remote areas, they have demonstrated adaptability through innovative solutions like efficient data management, vaccination awareness campaigns, and leveraging technology for improved care delivery. The findings are pertinent for not only health care practitioners and policymakers but also the broader scientific and public health communities. However, the findings may have limited generalizability beyond Manipur and Nagaland.

## Introduction

### Background

The foundational principle of public health in India hinges on the provision of equitable health care services, particularly in geographically challenging terrains [1]. Illustrating these geographical complexities is the North-Eastern region of India, nestled in the eastern Himalayas, presenting unique and formidable obstacles to health care delivery. Compounded by the proximity to international borders in the North-Eastern States, the health care landscape becomes even more intricate [2]. This geographical complexity leads to the isolation of many communities [3,4], significantly impeding access to health care services [5]. Consequently, these intricate geographical factors render the task of acquiring health care services daunting and often insurmountable for numerous isolated communities.

To deliver essential medical services in North-East India, health care workers grapple with a multitude of challenges [1,3]. These include logistical hurdles, such as navigating treacherous routes to reach rural villages. Transportation issues, exacerbated by the region’s geography and limited road infrastructure, further impede the mobility of medical staff and supplies. Adding to the complexity, North-East India boasts a diverse array of cultures and languages [4]. Geographical distance poses a significant barrier to health care access, especially in rural areas with poor transportation infrastructure, where nonmotorized transportation is common [6]. Health care professionals must adeptly tailor services to local cultural nuances, ensuring effective communication and cultural sensitivity [7] for interventions that are not only accessible but also relevant and accepted (eg, culturally respectful and patient‐centered care, healthy lifestyle promotion, increased family and community support, technology use for efficient and timely care, and increased disease knowledge).

Recognizing the intricate challenges faced by health care workers in these demanding terrains is crucial [4]. Limited access to timely medical interventions can lead to delayed disease management, increased disease burden, and preventable mortality [8]. Some prevailing issues in the health care sector include a shortage of trained personnel [9,10], addressing the needs of sparsely populated and remote areas, enhancing governance in the health sector, improving the quality of health services, optimizing existing facilities, ensuring effective and timely use of financial resources [11], and grappling with a high incidence of HIV/AIDS in Nagaland and Manipur [12,13]. Although the health care infrastructure has seen significant enhancements since the implementation of the National Rural Health Mission (NRHM) in 2005, the availability of specialists and trained health care workers remains a challenge [10].

Practically all primary health care centers (PHCs) and subcenters in the North-Eastern region lack good health care infrastructure compared with the national average [14]. The health care infrastructure in the North-Eastern region lacks medical professionals, nurses, and other staff, all of whom need to be hired and properly trained [9,15]. The lack of cold chain points, particularly in remote areas, creates logistical complexities, and the absence of training programs further hampers efficiency. Communication challenges during adverse weather conditions also affect decision-making. The absence of designated vehicles for vaccine transportation in remote areas forces health workers to travel to Imphal, causing in-person delivery. Geographical challenges also pose obstacles to medical supply [16]. Several studies have also identified increased workload and shortage of manpower [10,11].

Globally, rural health faces challenges primarily related to access, resource distribution, and shortage of health care professionals. Access to health care is a major issue in rural areas worldwide, with resources often concentrated in urban centers. Shortage of doctors and health professionals in rural regions contributes to the health care disparity [17]. Challenges in health care access in the Circumpolar North include the small size of community populations leading to limited local health care professionals, extensive travel requirements causing financial and emotional stress, and the potential need for physical relocation for treatment [18].

### Challenges During the COVID-19 Pandemic

Technical efficiency to evaluate how effectively resources like public expenditure are used to combat challenges, such as the COVID-19 pandemic, in health care systems, such as Manipur and Nagaland, is low due to the unique geographical and socioeconomic contexts of these regions [19]. During the COVID-19 pandemic, health care workers faced direct attacks, limited emergency services, and increased health care spending [20]. This led to higher treatment costs and inflation, affecting household income significantly. Shortage of resources, prolonged treatment, and increased hospitalization costs further exacerbated the challenges faced by workers in Nagaland [21]. The pandemic has highlighted the need for improved efficiency in health care service delivery [20,21].

### Objectives

This study aims to identify the challenges faced by health care workers in the rough terrains of various districts in Manipur and Nagaland, including transportation challenges. It also seeks to document the perceptions and awareness among health care providers regarding the COVID-19 vaccine and the pandemic situation, along with identifying the alternatives or actions adopted by health care workers to address these challenges. The main objective of the study is to record the reported challenges of health care workers who work at different levels such as implementation, management, and policy.

## Methods

### Participants

**Figure 1:**
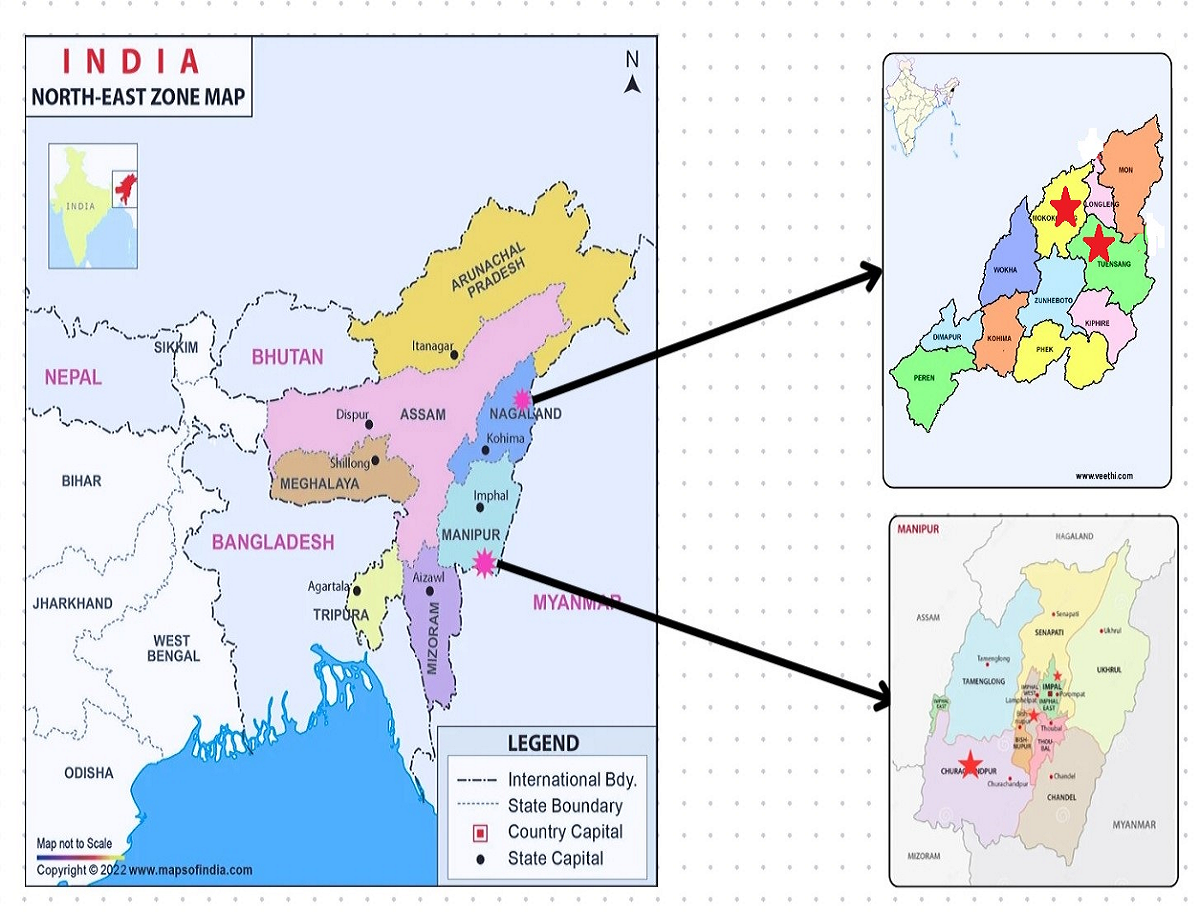
Pictorial representation of the 5 districts selected in Manipur and Nagaland in the North-East region of India. Health care workers were selected with purposive sampling and were interviewed in 2021.

A total of 29 health care workers from various health care facilities, including 4 district hospitals, 3 community health centers (CHCs), and 7 PHCs, from districts in Manipur and Nagaland were selected (Table 1 and Figure 1). This diverse sample aimed to capture variations in work culture and challenges across different health care settings.

**Table 1:** Age of health care workers according to gender, who were selected with purposive sampling and interviewed in 2021 from 5 districts in Manipur and Nagaland.

State and gender			Respondents^a^ (N=29), n (%)		Mean age (years)		Age range (years)
**Manipur**							
	Female	7 (24)		42.9		34-54	
	Male	12 (41)		44.7		32-58	
**Nagaland**							
	Female	6 (21)		35.8		24-52	
	Male	4 (14)		36.5		28-46	

^a^Among the respondents, 55% (16/29) were working at the implementation level, 24% (7/29) were working at the management level, and 21% (6/29) were working at the policy level.

### Inclusion Criteria

The study included all levels of health care professionals who were in active employment for the past 6 months without taking a significant vacation. Individuals who were engaged in ground-level implementation, policy, and maintenance activities were considered.

### Recruitment

Five districts were chosen after taking into account the various topographies of the region. Two districts were from Nagaland (Mokokchung and Tuensang), and three were from Manipur (Imphal West, Bishnupur, and Churachandpur) (Figure 1). The districts of Nagaland were selected because these are primarily covered with dense forests and mountainous terrain, while the districts of Manipur feature a physical environment made up of mountains, islands, valleys, and flatlands. Nineteen health facilities, including PHCs, CHCs, and district hospitals, from these 5 areas were selected for the investigation.

### Ethical Considerations

Prior to commencing the research, ethical clearance was obtained from the Indian Council of Medical Research (ICMR), Central Ethics Committee on Human Research (CECHR), Bengaluru, India (reference number: CECHR-001/2021). Additionally, all regulatory approvals from various stakeholders were obtained before starting the study. Informed consent was obtained verbally before participation. The consent was audio-recorded in the presence of an independent witness. The consent was voluntary, specific, and offered without coercion, bribery, or misinformation of any kind. Data collected from health care workers who were interviewed were deidentified. The participants have not been identified in any images of the manuscript or supplementary materials.

### Study Setting

This study is part of the “i-DRONE” (ICMR’s Drone Response and Outreach for North East) project conducted in the North-Eastern States of Manipur and Nagaland from 2021 to 2022, focusing on interventions related to health care personnel assisted by a drone for medical delivery. This study addresses the secondary objective of the i-DRONE project. The primary data for this study were obtained from health care workers actively involved in various aspects of health care delivery.

### Study Design

A purposive sampling technique was employed to select participants engaged in the universal immunization program (UIP), specifically those involved in vaccine and medical delivery, vaccine inoculation, and case management. The inclusion criteria were set for health care workers under the UIP and those willing to participate in one-on-one interviews. After selecting the staff, the researchers explained the study’s purpose and voluntary nature, and the assurance of confidentiality and anonymity.

### Measures

This descriptive study used a qualitative research approach to gain an in-depth understanding of the challenges experienced by health care workers engaged in service delivery at various levels of health care centers during the pandemic. The enquired probes (questions designed to gather data or insights) were selected, and researchers recorded the challenges faced by health care workers in the vaccine or medicine supply chain system during the pandemic (Table 2). Phone recorders were used to collect data. Semistructured questionnaires were used for in-depth interviews, which were conducted in November 2021 in face-to-face interactions by a member of the study team individually in English, Hindi, Meitei (Manipur), or Ao (Nagaland), and participant privacy was maintained. Interviews were conducted by 3 investigators (2 scientists and 1 research assistant) at the time of data collection. A local field investigator was present to assist in interviews conducted in Meitei and Ao, as the main interviewer was not aware of the local languages. Interviews were conducted by the main interviewer. Interviews lasted between 20 and 43 minutes, with an average of 27 minutes. Data were gathered up until the point of saturation. There was no prior relation of the research investigators with the participants. However, rapport establishment was the first step prior to the interviews.

Some of the interview questions were as follows: What is your role in the immunization program? As you have been engaged in vaccine delivery/inoculation procedures, what challenges you have encountered during the process? What are your thoughts on the availability of vaccines in your designated area? Apart from vaccination, what were the challenges you faced in managing the requests for other medical supplies and managing the caseloads? What are your thoughts about the intake of vaccines (good, effective)? Are you aware which vaccines have been approved by the health authorities in India? Before vaccination started, what were the challenges you were facing in the management of COVID-19 cases? Did you face a shortage of medicines? What are your views on the current vaccine delivery mechanism? Do you have any suggestions for improving the supply of vaccines, especially in remote areas?

**Table 2:** Various domains and probes (awareness and challenges related to service delivery encountered by health care workers) selected for the interviews conducted among health care workers selected with purposive sampling and interviewed in 2021 from 5 districts in Manipur and Nagaland.

Domain^a^	Probe^a^
Challenges associated with logistics and supply chain	Terrain (challenging roads), infrastructural issues, and delivery and execution
Difficulties in duty or job execution during the pandemic	Family issues during the pandemic, job transfer, mental exhaustion, and duty hours
Awareness and perception regarding COVID-19 vaccines	Availability of different types of vaccines and perception of the effectiveness of the vaccines
Challenges in service delivery	Infrastructure for patient isolation and sample collection, crowd management, panic situations, increased workload and erratic work hours, and stigma and discrimination

^a^Domains and probes were selected based on a literature review prior to conducting interviews with health care workers.

### Analysis

The data were analyzed with a thematic analysis approach using NVivo 14 (QSR International), which involved identifying and analyzing patterns and themes within the data. This analysis was used to summarize important elements of data collection since it is an organized way to handle and organize data to find themes and patterns. The classification by Braun and Clarke [14] was followed for the data analysis.

#### Data Familiarization

Each interview was recorded and later translated and transcribed verbatim for analysis. Researchers read and assessed the interviews to ensure that the data were correctly transcribed. Since the respondents spoke English, Manipuri, and Nagamese, the transcripts in the local languages were backtranslated into English and tallied by the researchers to make sure that the meaning remained unchanged. Data were cross-checked by other researchers to become familiar with the data.

#### Developing Codes

**Figure 2:**
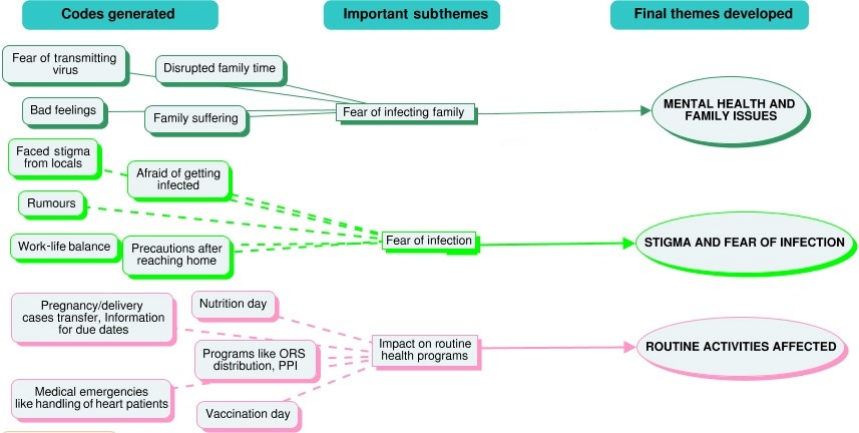
Generated codes, subthemes, and related final themes as an example of code generation for the data collected from health care workers selected with purposive sampling and interviewed in 2021 from 5 districts in Manipur and Nagaland.

After examining the transcripts, labels and codes were created (an example is provided in Figure 2) to cover the concepts based on participant responses, allowing researchers to become comfortable with the data. These transcripts were then coded independently by the researchers. Researchers having academic backgrounds in anthropology and community medicine identified the possible themes. The data were analyzed and the themes were framed within these constructs with illustrative quotes selected for each of the themes.

#### Thematic Review and Link Mapping

The themes that were developed addressed the study questions and provided insights into the interview data. Based on the principles of qualitative research, the study findings were categorized into common themes that emerged most frequently, and the salient themes were important but were reported less frequently by the respondents. Since many of the initial codes were shared or common to multiple themes, a thematic map was created in the preceding step of data analysis.

#### Review of Potential Themes

The themes reflected the interview data and answered the research questions. The thematic map constructed in the previous phase of data analysis was refined as several initial codes were shared or common to more than one theme.

#### Defining and Naming Themes

Themes were carefully reviewed in depth. Themes were refined, and appropriate names were assigned to each theme.

#### Writing a Thematic Narrative

The themes were then described in detail, drawing upon narratives from various subthemes to provide a comprehensive understanding of the participants’ perspectives and experiences.

As the interviews were followed by the semistructured questionnaire, investigator triangulation was performed with 2 anthropologists and 2 public health experts who provided their expertise to reduce the bias and enhance the credibility of the data. After going through all the data and interviews, researchers selected relevant interviews. Interviews where the conversation was not relevant were excluded. Finally, the codes and themes were refined after discussions with team members on variations in the themes.

## Results

### Overview

This section presents an overview of the perception and awareness of health care workers; the challenges faced in the health care system, especially in the context of vaccine distribution and administration in Manipur and Nagaland; and the adopted alternative ways of action to tackle those challenges.

### Challenges Reported by Health Care Workers

To describe the challenges, themes and subthemes were used, and relevant excerpts from the transcripts that represent the challenges faced by health care workers are presented in Textbox 1.

**Themes and subthemes for challenges reported by health care workers selected with purposive sampling and interviewed in 2021 from 5 districts in Manipur and Nagaland.**

1. General challenges
  - Challenges due to being an international borderline district
  - Human resource constraints
  - Logistical challenges of medical supply (most stated challenge)
  - Infrastructural issues
  - Transportation-related challenges (most stated challenge)
2. Challenges faced during the pandemic
  - Increased workload and disordered work hours (most stated challenge)
  - Lack of diagnostic centers
  - Mental health and family
  - Routine health care facilities affected
  - Stigma and avoiding contact with health care staff or workers
  - Vaccine uncertainties and misinformation
3. Perception and awareness regarding COVID-19 vaccination
4. Alternative actions or strategies adopted by health care workers to address the challenges
5. Suggestions provided by health care workers

### General Challenges

Health care workers face general challenges on a regular basis during work. These challenges do not include other factors. Some of the subthemes are described below.

#### Human Resource Constraints

During the pandemic, most of the medical staff said that they had additional responsibilities in handling the work due to which they felt burdened, such as personally delivering vaccines to PHCs and subcenters. One respondent made the following statement:

There is a lack of manpower… I have the job responsibility of maintaining a log of vaccine stock… but, now, I have to drive to PHCs, sub-centers and give them vaccine stock myself…. This is additional work, and I am spending fuel, money and also time in this.

This strains resources, including fuel and time, impacting overall efficiency. Training for health care personnel in both patient care and administrative responsibilities and additional manpower will be beneficial. The absence of training programs hampers the ability of staff to handle patient care and manage administrative tasks effectively.

#### Infrastructural Issues

Health care workers said that poor infrastructure in remote locations creates communication hurdles and delays in feedback due to adverse weather conditions. One respondent made the following statement:

When the network goes down, we do not receive any feedback from the cold chain points [state vaccine stores] as a result of which we have no access to their real-time data. We usually experience a delay of 24-48 hours.

Health care workers in Manipur face significant challenges related to weather conditions, particularly during the prolonged monsoon period lasting approximately 8 months.

#### Logistical Challenges of Medical Supply

A district logistics manager reported that due to high demand for essential supplies like masks, hand sanitizers, and personal protective equipment (PPE), improper distribution caused uneven access to resources that hampered health care delivery.

A lot of demands…for masks, hand sanitizers, PPE, head covers... No proper management of supplies from CHC, PHCs in terms of indent requests.

Distributing vaccines to remote areas presents significant transport challenges, including extended travel times and additional arrangements. Health care workers face challenges due to the uneven distribution of cold chain points and a lack of vaccine carrier boxes. A medical officer (MO) from Karang made the following statement:

...no cold chain point in Karang, therefore, we have one designated vaccination day, on which we conduct the vaccinations. Our officials collect the vaccines in the morning from District Family Welfare Office (DFWO), Bishnupur and then come to Karang…in the evening while returning, they will give the unutilized doses and vials back to DFWO office on same day… till now we are working with this mechanism.

This quote depicts that the labor-intensive nature of manual processes and travel between locations limit vaccine distribution efficiency.

#### Transportation-Related Challenges

Monsoons exacerbate other issues like infrastructure, making it difficult and dangerous to navigate poorly constructed roads, especially in remote areas.

During monsoon, it becomes very difficult to manage everything, as the road condition is very bad and it becomes dangerous.

Accessibility to several locations becomes worse during the torrential monsoon, which lasts for over half a year. The unpredictable weather in the regions, including continuous rain and frequent landslides, complicates transportation and access to remote areas. The combination of bad road infrastructure, challenging weather conditions, and geographical layout hampers health care delivery, making it time-consuming and difficult. According to health care workers, long-distance delivery, particularly in Sangang, is a significant challenge in procuring medical supplies and distributing vaccines. The 35-km distance between district hospitals and Churachandpur, coupled with unmetalled roads, results in a lengthy 2-hour journey. Sangang’s dependency on Imphal for medical supplies also introduces delays in obtaining stock, affecting the region’s ability to respond swiftly to health care needs. An auxiliary nurse midwife (ANM) stated the following:

The staff from PHC have to go to Imphal to get the vaccines on their own, ... carried in a two-wheeler, so, we have to be very cautious that the vaccines do not get damaged by jerks or vibrations...

Thus, transportation safety protocols are crucial to ensure vaccine safety and integrity during transit. Connectivity issues, including reliance on boats, add to the difficulties faced by patients in severe health crises.

On the way to Bahiang, there is a river, ... there is an old suspension bridge... During the monsoon, the river is flowing above the warning sign…so it is very difficult and scary to go across it using that bridge.

One respondent made the following statement:

...for delivering the vaccines from the Chief Medical Officer (CMO) office in Mokokchung to different cold chain points across Mokokchung but we are allotted limited funds...

Therefore, the time-consuming allocation of funds to staff and the time-consuming process of managing fuel and vehicle maintenance pose challenges for health workers.

#### Challenges due to Being an International Borderline District

The risk of ambush during transportation poses a threat to the safety of health staff, deterring individuals from willingly undertaking journeys to remote areas. One respondent commented as follows:

Recently, there was an ambush, and the convoy of our staff was going to Singngat... on the same day as the police had imposed curfew in that area. So, now, the staff are reluctant to go to such areas, because they consider it unsafe....

Moreover, security concerns are exacerbated by the proximity to international borders, as seen in Churachandpur district, which shares borders with Myanmar. This proximity hinders distribution drives as mentioned by a staff member:

...there are few insurgent activities which often hamper our distribution drives, as there are curfew and bands for safety...

The compounded issue of disrupted communication during bans or heavy rains adds an additional layer of fear among health care workers operating in these distant and insecure locations.

...there is a problem with communication…when there is curfew or heavy rains, phone signals also get hampered, which adds to fear among staff more.

### Challenges Faced During the Pandemic

The study was conducted in 2021, when the COVID-19 wave still prevailed; thus, health care workers mentioned various challenges that were related to COVID-19 in addition to the general challenges.

#### Mental Health and Family

The fear of transmitting the SARS-CoV-2 virus to family members is a prominent aspect, with health care workers (mostly females) expressing concerns about potential contact with COVID-19 patients during their daily work. One respondent made the following statement:

We were afraid of getting infected or transmitting the virus to our family members.

This fear is compounded by challenges in convincing the local population to take the vaccine due to a lack of awareness. A senior female staff nurse from Karang commented as follows:

But from Karang island, we have to go in shared boats and vehicle… so, I felt always exposed to infection … I was scared and thus, initially, I was self-isolating myself from my family. COVID-19 has disrupted family time.

The meticulous sanitation rituals before leaving work and upon reaching home underscore the heightened anxiety around preventing infection transmission. The overarching theme captures the delicate balance health care workers navigate in terms of their professional responsibilities, their fears of virus transmission, and the impact on their mental well-being and family life during the COVID-19 crisis. One female health staff (FHS) member stated the following:

My family also suffered from stigma because of me and I feel bad…although, I was doing my duty of attending to patients.

This statement depicts the fear of health care workers for their families.

#### Stigma and Avoiding Contact With Health Care Staff or Workers

Experiences of stigma have been reported by health care workers who were engaged in COVID-19 duties and vaccination campaigns. One respondent stated:

People used to think that since we are working in COVID-19 management, we will transmit infections to them...

Respondents from both the states said that villagers in small hamlets had banned entry of any health care worker in the area as they believed that doctors, nurses, and others involved in the management of COVID-19 cases would bring the infection to the village and infect all villagers. One respondent made the following comment:

10 km from here village populated with 100 individuals, currently, no one is vaccinated as they do not let medical teams enter the premises.

The nurses who were staying in those villages were ignored and compelled to stay outside the village (using hospital quarters).

#### Routine Health Care Facilities Affected

The excerpt highlights the significant challenges and disruptions faced by routine health programs due to various factors, primarily the impact of COVID-19 and administrative issues. A female health worker made the following statement:

Often the parents of the children miss out on coming on the due date of the vaccination, the Accredited Social Health Activist (ASHA) worker often informs them priorly regarding the due date and special immunization drives. For polio, right now… conducting house to house program apart from the designated polio day. We also conduct Nutrition Day once a month program where we will go to villages which come under PHC Phayeng and distribute vitamin supplements, ORS, medicines for children and mothers, however, due to COVID-19, right now this activity is on hold.

Administrative issues further compound the problem, as infrastructure remains nonfunctional, hindering the initiation of essential services like assisted deliveries and emergency care for heart attacks or snake bites.

#### Increased Workload and Disordered Work Hours

All respondents, including stakeholders, indicated that increases in workload and work responsibility have been major challenges during the pandemic. They were assigned multiple managerial tasks, including record keeping, stock maintenance, requisition submission, crowd management, and distribution of medical kits.

...the crowd management was a big challenge, in which we took help of the security and police.

This workload affected their work-life balance, and female respondents worried about their families feeling neglected due to long-shift jobs. A district immunization officer (DIO) made the following statement:

Since COVID-19 has started. We have not been able to take leaves for any festival… We have been continuously working since 1st March 2020 without any leave for personal family time or vacation.

They also faced challenges in handling crowds during mass vaccination drives, as they had to maintain COVID-19 protocols and adopt standard operating procedures due to poor infrastructure in remote health care facilities.

#### Vaccine Uncertainties and Misinformation

Initially, fear and apprehension about vaccination were alleviated by health care workers who remained healthy after receiving the vaccine. However, misinformation and hesitancy led to delayed diagnosis and treatment, increasing the risk of contamination. An ANM commented as follows:

Many people were afraid as they got some fake messages on WhatsApp and other places.

In response to the first wave of the pandemic, the medical director in Imphal allowed the MO in-charge to request sample collection for 10 or more symptomatic individuals requiring testing.

#### Lack of Diagnostic Centers

A lack of sample collection and testing centers in all districts also posed challenges in managing severe acute respiratory infections. A senior nurse (SN) from Karang said the following:

Delay in sample collection in Karang island, Manipur... led to delayed diagnosis and till that time there was high risk of contamination…

Thus, delay in diagnosis and treatment heightened the risk of contamination. The urgency of having local sample collection facilities has been emphasized, as seen during the first wave when sample collection sites were centralized in Imphal.

### Perception and Awareness Regarding COVID-19 Vaccination

The participants were probed about their perception and awareness regarding the COVID-19 vaccine. An ANM made the following statement:

Covishield vaccine is good for lessening COVID cases. People who took vaccine had mild symptoms and recovered in 2-3 days. Due to vaccine, the caseload decreased and recovery of people was faster.

It was observed that all health care officials and stakeholders were aware of the names and types of COVID-19 vaccines available in India. The majority of participants (89%) named the Covishield vaccine only, as it was the only COVID-19 vaccine available in North-East India at the time of the study, and the remaining health care workers mentioned the names of vaccines, such as Covaxin and Sputnik-V, in their responses, which were approved for emergency use in India. Participants in a study expressed a positive attitude toward the COVID-19 vaccine, stating that vaccinated individuals experienced mild symptoms after infection. They also noted higher COVID-19 cases among nonvaccinated individuals. Health workers initially faced hesitancy to take the vaccine, but they played a crucial role in encouraging it. The vaccine reduced cases and reduced the health care burden. Public awareness programs have been ongoing to motivate people about the benefits of vaccination. Despite regional differences in vaccine availability and specific vaccines, there is a consistent positive perception of vaccine effectiveness. Government approvals and regulations are acknowledged, and vaccination coverage varies.

### Alternative Actions or Strategies Adopted by Health Care Workers to Address the Challenges

During the COVID-19 pandemic in Manipur and Nagaland, health care workers demonstrated remarkable adaptability in overcoming logistical challenges and vaccine shortages. When faced with difficulties in medical supply logistics, health care workers took matters into their own hands by personally picking up and distributing supplies to regional centers, ensuring uninterrupted health care services.

Initially, we used to pick up the vaccines from the main head office in Dimapur…But we are lucky that the medical officers of the health centers are very cooperative that they come up, pick the vaccine and take it there.

DIO4

To prevent vaccine shortages, health care workers proactively preordered stocks for remote PHCs and subcenters, averting potential crises and ensuring continuous vaccination efforts. One district vaccine logistics manager (DVLM) commented as follows:

As since, we get all the supplies from the capital city i.e. Imphal which is situated approx. 50 km from here, it was not possible to get stocks immediately. Thus, we used to pre-order as ours is the hub from where all the cold chain points in CCPur get the supply.

DVLM2

The use of online dashboards enhanced operational efficiency, enabling real-time data management and streamlined decision-making amidst resource constraints.

Online dashboard shows the stock at the cold chain points. So, we used to get notification from there on the stock of vaccines at different health centers. We have to then relocate these stocks from the areas where these are available.

DIO4

Despite insufficient human resources, health care workers multitasked effectively. ANMs engaged in routine immunization and data management, and conducted awareness programs, vaccination camps, and home visits, exemplifying their commitment to comprehensive community health care. The increased workload prompted innovative approaches. Health care workers actively engaged stakeholders, including church priests, village councils, and administrative bodies, in vaccination awareness programs, fostering community cooperation, which was crucial for successful immunization campaigns.

We conducted awareness camps, taken help of church priest, village council, administrative, sensitization, we have done everything, but people in these villages are very frightened and do not want to take the vaccine.

DIO4

Implementing “har ghar dastak” initiatives, health care workers conducted house-to-house visits to enhance vaccine acceptance and deliver essential health education, mitigating vaccine hesitancy exacerbated by misinformation.

We are taking help from village council, heads, church. The church leader says we will do that but still there is some resistance. We are conducting har ghar dastak campaign sessions in 18-20 villages.

DIO4

Patients requiring isolation and medication received dedicated care, with health care workers swiftly shifting individuals to isolation wards and providing home medication kits, facilitated by collaboration with accredited social health activists. Vaccine awareness camps organized by ANMs further addressed community concerns, ensuring informed decision-making and reinforcing trust in vaccination efforts.

### Suggestions From Health Care Workers

Looking forward, health care workers offered insightful suggestions to enhance future preparedness and response strategies. To tackle logistical challenges, they advocated for expanding cold chain infrastructure, increasing cold chain points at remote PHCs and subcenters, and equipping facilities with essential accessories like temperature loggers and generators to maintain vaccine efficacy.

...since remote locations may get cut-off because of the weather conditions, there should be ample cold chain points for storage of vaccines in bulk in such times.

DVLM2

Embracing digital solutions for record-keeping and inventory management emerged as a priority, aimed at improving efficiency in supply chain logistics and laboratory facility management.

I am constantly saying that digitization is important. By that any person can get to know that what stocks are available and what is not. It will make record keeping easier.

MO2

Addressing infrastructure vulnerabilities, health care workers emphasized the need to introduce 4×4 vehicles to mitigate monsoon impacts on road conditions to ensure timely access to remote villages, which is critical for health care delivery during emergencies.

Including fleet of transport varying from different size and capacity will be useful, as not all places can be accessed by big vaccine carrier vans. They have provided us 4×4 vehicles this time for har ghar dastak, which is better than those vans in terms of stability and access.

DIO1

To address insufficient human resources, health care workers recommended capacity-building initiatives tailored for specific supply chain roles, enhancing operational resilience and workforce effectiveness.

We have a lot of manpower in the district hospital, however, currently in COVID scenario, they are overburdened with caseloads, management of patients and vaccine inoculation. In my suggestion, there shall be some extra staff, who can be technically trained for this purpose only. So, it will give employment and also be supporting to the staff member.

DIO2

They also emphasized designing a proper road map for the supply chain with the inclusion of designated members to reduce the workload on existing staff.

A proper road map for supply chain shall be designed with designated members for carrying out work, instead of putting burden on the existing staff will be useful. This is lessening the burden of work on the staff and also, generate opportunities for more people in terms of capacity building.

MO1

In remote areas, improving transportation mechanisms and storage conditions for vaccines and possibly exploring drone technology emerged as crucial steps to safeguard vaccine integrity and accessibility.

Otherwise, right now it is very difficult to transfer the pregnant females through boat to BPR. Installation of a cold chain point for routine and COVID immunization will be really helpful, as this will also save time. Provision for a dedicated transportation facility for staff and vaccines and other medical materials from Thanga, BPR, and Imphal shall be put in place.

SN1

The state team will have better idea regarding this. In majority of Manipur vaccines are delivered through cars or vans or boat. The integration of drone technology into the existing system will improve the connectivity and our workload will also reduce in an effective manner.

ANM1

It will be beneficial for hilly areas, sake of our state and whole of north east if drones are successful.

ANM2 Phayeng

Developing emergency procurement divisions for rapid medical supply acquisition from nearby centers during crises was also highlighted to quickly enhance the response.

As you are now aware of the remoteness of our location, in case any provision can be made that we can procure medical supplies in cases of emergency from nearby CHC, PHC and DH in BPR instead of CCPur for efficiency and time.

DIO3

Lastly, combating vaccine hesitancy and misinformation required sustained efforts in awareness and communication, particularly among tribal populations residing in remote and inaccessible areas, underscoring the importance of culturally sensitive health care outreach strategies.

There is less awareness among tribal people and thus, we need to work on spreading awareness among them especially those who are living in interiors.

FHS1

## Discussion

### Principal Findings

During the COVID-19 pandemic, health care workers in Manipur faced direct attacks on ambulances owing to conflicts that arose, which directly hindered emergency services and indirectly affected routine health services due to the prevailing insecurity among the population [20]. This study depicted the challenges related to the logistical management of medical supplies and the routine activities of programs such as vaccination. Health care inflation was not observed in this study, but a previous study stated that high treatment costs impacted household income in Nagaland [21]. There was a shortage of health care resources in this study, including a shortage of manpower and 4×4 vehicles. The COVID-19 pandemic posed significant challenges that disrupted routine health programs, causing missed vaccinations, adjustments in immunization strategies, and temporary suspension of essential programs [22]. One subtheme in this study that came to light was the absence of diagnostic facilities, which had a major impact on the management of severe acute respiratory infections owing to the insufficient distribution of sample collection and testing centers among districts. During the first wave, Imphal’s centralization highlighted the urgent need for decentralized local institutions to speed up diagnosis and treatment, lowering the danger of contamination. However, the number of diagnostic centers in the region needs to be increased. Studies have shown that excessive work pressure causes mental distress, weakness, and anxiety among health care workers [23]. The findings of this study are in line with those of previous studies showing that health care workers have faced increased workloads and responsibilities, leading to mental health strain and a lack of vacation leaves. The responses of health care workers in our study indicating the fear of infecting their family members and the sadness of staying away from their own children coincided with the findings in a multicentric study conducted in India among health care workers during the pandemic [24]. Additionally, the mental health challenges indicated that women in health care service delivery were facing triple burdens [25] as they were engaged as caregivers in health centers, were engaged in home activities, and had to balance their mental well-being and their family’s health [26]. Several studies identified cases of stigma during the COVID-19 pandemic as challenges among health care workers [27-29]. Similarly, this study found that community stigma and the fear of getting infected led to exclusion and discrimination. To mitigate these challenges, it is essential to introduce awareness programs and capacity building for additional staffing, clearly delegate responsibilities, and introduce policies supporting work-life balance. Misinformation, particularly through social media, has also contributed to public hesitancy toward vaccination. Health care workers face significant problems due to inadequate infrastructure in distant places. This can hinder communication and cause delays in feedback, which can be made worse by unfavorable weather conditions. Workers in Manipur have to deal with an extended monsoon season that lasts for roughly 8 months. This makes it more difficult to provide services, which exacerbates transportation problems. It is also dangerous to navigate on damaged roads in remote areas, which is made worse by frequent rain and landslides lasting for more than 6 months. This hampers timely health care delivery, and this is particularly evident in Sangang where a 35-km journey to Churachandpur takes 2 hours owing to bad road conditions, impacting medical supply distribution and vaccine accessibility. Improving infrastructure, optimizing vaccination strategies, and involving law enforcement in crowd control are essential for managing the pandemic and ensuring the safety of health care workers. The study found that health care workers had a good understanding of COVID-19, with the Covishield vaccine being the most popular choice. Despite initial hesitancy, they played crucial roles in encouraging vaccination and fostering trust in the community through awareness programs. Postvaccination campaigns have led to a decline in cases and have reduced the health care facility burden. Despite regional differences in vaccine availability, there is a shared confidence in the protective nature of vaccines. The findings of this study align with those of a knowledge, attitude, and practice study conducted among health care workers in India, which found that over 80% of them had adequate knowledge, a positive attitude, and safe practice regarding the infection [30].

The COVID-19 pandemic highlighted the adaptability and resilience of health care workers in Manipur and Nagaland, who faced significant challenges while implementing alternative strategies. Throughout the pandemic, health care workers used a variety of innovative approaches. ANMs played crucial roles in data management, immunization outreach, and organizing vaccination camps, alongside conducting home visits and ensuring patient isolation. Health care workers irrespective of their posts took on the responsibility of vaccine transportation using their own vehicles, facilitating effective supply chain management. Health care workers demonstrated a solid understanding of COVID-19 infection and vaccine efficacy, with the Covishield vaccine being the preferred choice owing to its proven effectiveness in reducing severity. Collaborative efforts with stakeholders promoted vaccination awareness and efficient crowd management, which are crucial for mitigating the spread of the virus. Initial vaccine hesitancy was addressed through community awareness programs led by health care workers and supported by accredited social health activists, leading to increased vaccination uptake and a subsequent decline in COVID-19 cases. Collaboration with local authorities, implementation of safety protocols, and ensuring adequate security infrastructure were identified as critical for safeguarding health care workers during vaccine distribution efforts. The experiences of health care workers in Manipur and Nagaland underscore the importance of adaptive strategies, robust infrastructure, and community engagement for overcoming pandemic challenges, providing valuable lessons for future health care preparedness and response strategies.

Health care workers in Manipur provided insightful suggestions to address challenges in cold chain logistics for vaccine distribution. To improve vaccine supply, they recommended establishing a robust transportation mechanism with a focus on preventing spillage or harm to vaccine quality during transit. Increasing the number of cold chain points, especially in remote areas susceptible to weather-related disruptions, has been proposed to enhance accessibility and bulk storage during adverse conditions. In terms of infrastructure, health care workers advocated for fleet diversity, including vehicles of varying sizes and capacities, to navigate diverse terrains. They stressed the importance of improving overall infrastructure to minimize travel time, introducing 4×4 vehicles for stability during emergent situations, and expanding cold chain facilities in interior locations.

Overall, this study presented the challenges faced by health workers in Manipur and Nagaland, and alternative strategies were adopted by health care workers to address the challenges reported by them. Furthermore, participants provided suggestions to overcome the challenges, including strategic planning, investment in better road infrastructure, and providing necessary resources and support. Collaboration with local authorities, implementing safety measures, and providing necessary security infrastructure are essential for ensuring the safety and efficiency of health care workers in these challenging regions. The strengths and limitations of this study are presented in Textbox 2.

**Strengths and limitations of the study.**

**Strengths**

1. The study provides a detailed discussion of the challenges faced by health care workers during the COVID-19 pandemic in Manipur and Nagaland, covering various aspects such as mental health strain, vaccination strategies, coping mechanisms, and infrastructure needs.
2. The study considers the specific context of Manipur and Nagaland, acknowledging the unique challenges and circumstances faced by health care workers in these regions.
3. The study offers practical recommendations to address the identified challenges, such as improving cold chain logistics, enhancing transportation mechanisms, and strengthening infrastructure, which can guide policymakers and health care authorities in formulating effective strategies.
4. The findings of this study are consistent with those of previous studies, enhancing the credibility and relevance of the study’s conclusions.

**Limitations**

1. The findings may have limited generalizability beyond Manipur and Nagaland, as the study focuses specifically on these regions and may not represent the experiences of health care workers in other areas.
2. The study relies on self-reported data from health care workers, which may be subject to biases, such as social desirability bias or recall bias, affecting the accuracy and reliability of the findings.
3. The study may lack longitudinal data to assess the long-term impact of the pandemic on health care workers and the effectiveness of implemented strategies over time.
4. The study may have limitations in capturing the perspectives of diverse communities due to language barriers or cultural differences, as the Kuki population is large in the area.

### Conclusions

This study explored and elucidated the multifaceted challenges that confront health care workers in North-East India’s geographically challenging terrains. Through rigorous analysis and evidence-based insights, we attempted to contribute to the advancement of health care service delivery in this region, ultimately striving for better health outcomes for its diverse populations. By examining these impediments in detail, we endeavored to provide evidence-based insights that can inform policy decisions and interventions to enhance health care delivery, ultimately improving health outcomes in the region. This research is pertinent for not only health care practitioners and policymakers but also the broader scientific and public health communities. It sheds light on the intricate challenges faced by health care workers in delivering services to marginalized populations in geographically challenging terrains, offering a foundation for evidence-based strategies to address these issues. In summary, this study is a voyage into the heart of the challenges that confront health care workers in North-East India’s geographically challenging terrains. It is crucial to address mental health concerns and security concerns.

## Abbreviations

ANM: auxiliary nurse midwife
CECHR: Central Ethics Committee on Human Research
CHC: community health center
DIO: district immunization officer
DVLM: district vaccine logistics manager
FHS: female health staff
ICMR: Indian Council of Medical Research
i-DRONE: Indian Council of Medical Research’s Drone Response and Outreach for North East
MO: medical officer
PHC: primary health care center
SN: senior nurse
UIP: universal immunization program

## Data Availability

The data sets generated during and analyzed during this study are not publicly available because of the restrictions from the government but are available from the corresponding author upon reasonable request.
